# Identification of Gene Modules and Hub Genes Associated with *Sporisorium scitamineum* Infection Using Weighted Gene Co-Expression Network Analysis

**DOI:** 10.3390/jof8080852

**Published:** 2022-08-15

**Authors:** Zongling Liu, Xiufang Li, Jie Li, Haiyun Zhao, Xingli Deng, Yizu Su, Ru Li, Baoshan Chen

**Affiliations:** 1 State Key Laboratory for Conservation and Utilization of Subtropical Agro-Bioresources, College of Life Science and Technology, Guangxi University, Nanning 530004, China; 2 Guangxi Key Laboratory of Sugarcane Biology, College of Agriculture, Guangxi University, Nanning 530004, China

**Keywords:** weighted gene co-expression network analysis, transcriptome, *Sporisorium scitamineum*, hub genes, candidate effectors

## Abstract

*Sporisorium scitamineum* is a biotrophic fungus responsible for sugarcane smut disease. To investigate the key genes involved in *S. scitamineum* infection, we conducted RNA sequencing of sugarcane sprouts inoculated with *S. scitamineum* teliospores. A weighted gene co-expression network analysis (WGCNA) showed that two co-expressed gene modules, MEdarkturquoise and MEpurple—containing 66 and 208 genes, respectively—were associated with *S. scitamineum* infection. The genes in these two modules were further studied using Gene Ontology (GO) enrichment analysis, pathogen-host interaction (PHI) database BLASTp, and small secreted cysteine-rich proteins (SCRPs) prediction. The top ten hub genes in each module were identified using the Cytohubba plugin. The GO enrichment analysis found that endoplasmic reticulum-related and catabolism-related genes were expressed during *S. scitamineum* infection. A total of 83 genes had homologs in the PHI database, 62 of which correlated with pathogen virulence. A total of 21 proteins had the characteristics of small secreted cysteine-rich proteins (SCRPs), a common source of fungal effectors. The top ten hub genes in each module were identified, and seven were annotated as Mig1-Mig1 protein, glycosyl hydrolase, beta-N-acetylglucosaminidase, secreted chorismate mutase, collagen, mRNA export factor, and pleckstrin homology domain protein, while the remaining three were unknown. Two SCRPs—SPSC_06609 and SPSC_04676—and three proteins—SPSC_01958, SPSC_02155, and SPSC_00940—identified in the PHI database were also among the top ten hub genes in the MEdarkturquoise and MEpurple modules, suggesting that they may play important roles in *S. scitamineum* infection. A *S. scitamineum* infection model was postulated based on current findings. These findings help to deepen the current understanding of early events in *S. scitamineum* infection.

## 1. Introduction

Sugarcane is the most important sugar-producing crop worldwide. *Sporisorium scitamineum* (*S. scitamineum*) is responsible for sugarcane smut, a disease responsible for a dramatic loss of crop yield. Sugarcane plants infected with *S. scitamineum* exhibit tiny stems, slender leaves, and fibrous stalks and contain a lower sugar content [1]. The *S. scitamineum* life cycle has three distinct phases, haploid yeast-like sporidia (basidial spores), dikaryotic hyphae, and diploid teliospores [2]. In the disease cycle, dikaryotic teliospores deposited on the sugarcane sprouts undergo meiosis to develop yeast-like haploid basidial spores. When the basidial spores germinate, two compatible haploid sporidia with opposite mating types combine to form dikaryotic hyphae, which can infect the meristem tissue of the sugarcane sprout. After a period of latent infection, the dikaryotic hyphae differentiate, form teliospores within the plants, and are eventually released into the environment by a specialized structure composed of teliospores and plant tissues, which are termed whip [3].

To facilitate the infection, plant pathogenic fungi secrete effectors into the plant cells to suppress the host immune response [4,5]. A total of 68 effector candidates have been identified in the *S. scitamineum* genome [6]; while little is known about their roles in plants, only *SsPele1* and *SsPEP1* have been functionally characterized to date [7]. Additionally, the other five candidates, which contain characteristics of signal peptides, low molecular mass, and upregulation *in planta*, could affect programmed cell death (PCD) by using transient expression on *Nicotiana benthamiana* [8,9]. A lack of sufficient knowledge on effectors has prevented a complete understanding of the pathogenesis of sugarcane smut.

Weighted gene co-expression network analysis (WGCNA) is an efficient method for mining modules and hub genes related to traits of interest [10] and has been successfully used to construct gene co-expression networks in different species [11,12,13]. In this report, a WGCNA approach was used to identify modules and hub genes associated with *S. scitamineum* infection. These findings will help to advance the current understanding of sugarcane smut, particularly during the early stages of the infection process.

## 2. Materials and Methods

### 2.1. Biological Materials and S. scitamineum Inoculation

In the field, the sugarcane cultivar, Zhongzhe 1 (ZZ1), is smut-resistant, while the cultivar, ROC22, is smut-susceptible [14]. However, both varieties are readily infected with *S. scitamineum* through artificial inoculation using the root-dip method of tissue culture-derived plantlets, suggesting that physical barriers provide the major mechanism of resistance in ZZ1 [15]. The cultivars were grown in the field to provide matured stalks with at least 20 nodes. *S. scitamineum* teliospores were collected from whips on ROC22 that had been inoculated with JG35/JG36 haploid yeast-like sporidia. Sugarcane cuttings, each consisting of a single bud, were washed with flowing water and incubated at 28 °C and 90% RH for bud germination. When the sprouts reached approximately 2 cm, they were injected with an *S. scitamineum* teliospore suspension (5 × 10^6^ cfu/mL) [16]. The inoculated sprouts were kept at 28 °C and 90% RH. After 24-h, 72-h, and 168-h post-inoculation, the sprouts were sampled, immediately frozen in liquid nitrogen, and stored at −80 °C until further use. The equal mix of JG35 and JG36 haploid yeast-like sporidia were considered to be the control (named as 0 h). Each treatment involved three biological replicates.

### 2.2. Visualization and Quantification of S. scitamineum in Plants

The sugarcane sprouts were crosscut to obtain approximately 1 cm slices. The slices were soaked in hyalinized solution (lactic acid: ethylene glycol: ddH_2_O = 1:1:1) and transferred to a boiling water bath for 8 min for hyalinized processing. The hyalinized slices were stained with 0.4% trypan blue for 30 s and washed three times with ddH_2_O. The slices were then hyalinized again as previously described and observed with an Olympus microscope BX51 (Bartlett, TN, USA).

TaqMan RT-qPCR was used to quantify *S. scitamineum* in the plants. The total DNA of sprouts inoculated with *S. scitamineum* was separately extracted using PlantZol (EE141, Transgen, Beijing, China) according to the manufacturer’s instruction. The quantity and purity of total DNA were analyzed using Nanodrop (Thermo, Waltham, MA, USA) and agarose gel electrophoresis. The *S. scitamineum bE* gene, which plays a role in haploid sporidia mating [17], was used as a target to measure the *S. scitamineum* copy number in the plants. The primers were bEQ-F: 5′-TGAAAGTTCTCATGCAAGCC-3′ and bEQ-R: 5′-TGAGAGGTCGATTGAGGTTG-3′, and the TaqMan probe was 5′-FAM-TGCTCGACGCCAATTCGGAG-TAMRA-3′ [18]. The TaqMan RT-qPCR reaction mix included 10 μL of 2x PerfectStart^®^ II Probe qPCR SuperMix (AQ711, Transgen, Beijing, China), 0.4 μL of each primer (10 μmol), 0.4 μL of TaqMan probe (10 μmol), 1 μL of template DNA (100 ng/μL), and ddH_2_O to a total volume of 20 μL. The PCR reaction involved the following: 95 °C for 30 s, 40 cycles of 95 °C for 10 s, and 60 °C for 30 s. The *bE* recombinant plasmid, pCE2-*bE*, constructed using the 5-min TA/Blunt-Zero Cloning Kit (C601, Vazyme, Nanjing, China) according to the manufacturer’s instruction, was used to construct a standard curve. The statistics were conducted as described previously [18].

### 2.3. RNA Extraction, Library Construction, and Sequencing

Sugarcane sprouts were harvested and subjected to total RNA extraction using a TRIzol Reagent kit (Invitrogen, Carlsbad, CA, USA) according to the manufacturer’s instructions. After DNase I treatment, the purity and integrity of RNA were analyzed using Nanodrop (Thermo, Waltham, MA, USA) and agarose gel electrophoresis. The RNA was quantified using Qubit3.0 with the QubitTM RNA Broad Range Assay kit (Q10210, Life Technologies, Carlsbad, CA, USA). RNA (2 μg) was used to construct a sequencing library using the KC-DigitalTM Stranded mRNA Library Prep Kit for Illumina^®^ (DR08502, Wuhan Seqhealth Co., Ltd., Wuhan, China) and fragments ranging from 200 to 500 bp were enriched. Paired-end sequencing was performed on the Novaseq 6000 (Illumina, San Diego, CA, USA).

### 2.4. Analysis of RNA-Seq Data

Raw data were first filtered using software Trimmomatic v0.36 (Anthony M. Bolger, Berlin, Germany), and the reads with low quality or joints were removed. In-house Perl scripts were used to cluster reads according to unique Molecular Identifiers (UMI) for removing errors and preferences generated by PCR amplification. The reads were mapped to the *S. scitamineum* genome (GenBank: GCA_900002365.1) using software STAR v2.5.3 (Alexander Dobin, New York City, NY, USA). The values of the per kilobase per million mapped reads (RPKM) were calculated using FeatureCounts v1.5.1 software, and differentially expressed genes (DEGs) were analyzed using the R edgR package v3.12.1. The genes with a resulting *p* value smaller than 0.05 were considered as DEGs. All genes were annotated using NCBI non-redundant protein sequences (NR), Protein Family (Pfam), Swiss-Prot, and Clusters of Orthologous Groups of proteins (COG).

A Venn diagram was created using software TBtools v1.09 (Chengjie Chen, GuangZhou, China) [19]. A WGCNA of all the genes was conducted using the R WGCNA package [10], and the threshold was determined using the scale-free network principle. The genes were divided into different modules based on their expression pattern. The *S. scitamineum* samples from the ROC22 and ZZ1 cultivars infected for 24 h, 72 h, and 168 h were considered as 24 h, 72 h, and 168 h traits, respectively. The gene-module correlation was analyzed using the Pearson correlation. The hub genes in the modules of interest were determined using the Cytohubba pluggin [20] in Cytoscape v3.9.1. Gene Ontology (GO) enrichment analysis was conducted using TBtools v1.09.

### 2.5. Effector Prediction

Genes in key modules were selected for candidate effector prediction. First, the gene sequences were translated into proteins using TBtools v1.09. These proteins were subjected to BLASTp analysis against the Pathogen Host Interaction (PHI) database (http://www.phi-base.org/, accessed on 8 May 2022). The molecular masses of the proteins were determined using TBtools v1.09, the signal peptides were predicted using SignalP v4.1 (https://services.healthtech.dtu.dk/service.php?SignalP-4.1, accessed on 8 May 2022), cysteine content was calculated using DiANNA v1.1 (http://clavius.bc.edu/~clotelab/DiANNA/, accessed on 8 May 2022), subcellular localization was predicted using ProtComp v9.0 (http://linux1.softberry.com/berry.phtml?topic=protcompan&group=programs&subgroup=proloc, accessed on 8 May 2022), and transmembrane domains were predicted using TMHMM v2.0 (https://services.healthtech.dtu.dk/service.php?TMHMM-2.0, accessed on 8 May 2022). Proteins were considered candidate effectors if they met the characteristics of small secreted cysteine-rich proteins (SCRPs) [21]: (1) molecular mass <400 amino acid (AA) residues, (2) cysteine content >3%, (3) presence of an N-signal peptide, and (4) lacking a transmembrane domain.

### 2.6. Reverse-Transcription Quantitative PCR (RT-qPCR)

RNA samples used for RNA-seq were also used in RT-qPCR analysis. The qPCR primers were designed using the primer-BLAST tool (https://www.ncbi.nlm.nih.gov/tools/primer-blast/, accessed on 15 May 2022). Reverse transcription and qPCR were conducted using BeyoRT™ III cDNA synthesis premix (5X) (with gDNA EZeraser) (D7185M, Beyotime, Shanghai, China) and ChamQ Universal SYBR qPCR Master Mix (Q711, Vazyme, Nanjing, China) according to each manufacturers’ instructions. The housekeeping genes, inosine 5′-monophosphate dehydrogenase (S10) and SEC65-signal recognition particle subunit (S11), were chosen as internal standards in the qPCR analysis [22]. Each sample was replicated three times. Expression data were analyzed using the 2^−ΔΔCt^ method [23]. All primers were listed in Table S1.

## 3. Results

### 3.1. Colonization of S. scitamineum in Sugarcane Sprouts

**Figure 1:**
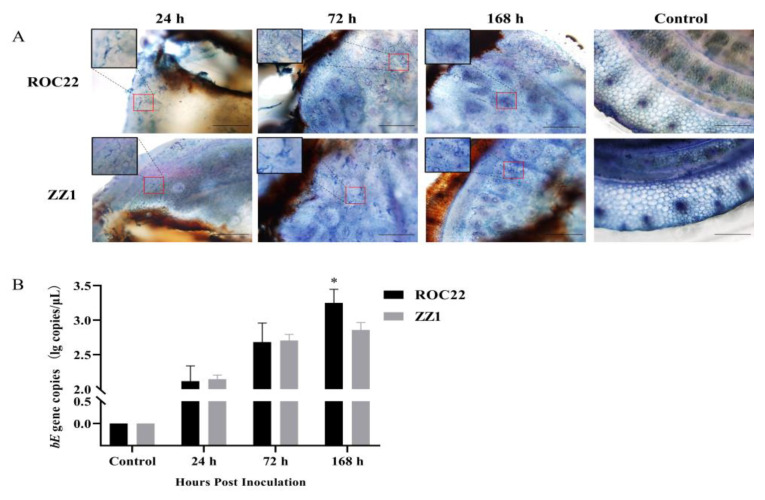
Visualization and quantification of *S. scitamineum* in sugarcane sprouts. (**A**) Sugarcane sprouts inoculated with *S. scitamineum* teliospores at different time points. The brown parts on the sprouts are the inoculation sites. The upper left corner is an enlarged view of the region indicated by the red box. Bars = 50 μm. (**B**) Quantification of *S. scitamineum bE* gene copies in sugarcane sprouts, and this gene played a role in haploid sporidia mating. The DNA contents of all samples were 100 ng. After 168-h post-inoculation, the number of *S. scitamineum* copies differed significantly between ROC22 and ZZ1 (* *p* < 0.05). The sprouts without inoculation were considered as controls. Three biological replications were conducted, and each replication contained five sprouts.

*S. scitamineum* induces systemic infection in sugarcane by colonizing the meristem tissue of the shoot [24]. To verify the infection by artificial inoculation, ROC22 and ZZ1 sprouts were checked by trypan blue staining for the presence of fungal hyphae in the meristem tissue. As early as 24-h post-inoculation, hyphae were present in both cultivars, and more were observed after 72 h and 168 h (Figure 1A). The standard curve of the *bE* gene was successfully constructed for further *S. scitamineum* quantification (*R*^2^ = 0.9953) (Figure S1). PCR quantification of the *S. scitamineum*-specific *bE* gene showed that the fungus proliferated rapidly in both cultivars for the first 72 h. However, by 168 h, the fungus was continuing to accumulate exponentially in ROC22, but had seemingly plateaued in ZZ1 sprouts (Figure 1B).

### 3.2. Analysis of RNA-Seq Data

RNA was sequenced from the *S. scitamineum*-inoculated sprouts at 24-h, 72-h, and 168-h post-inoculation and from the haploid *S. scitamineum* basidiospores. The combined sequences reached 230.24 GB of data and 1,534,886,652 raw reads, and Q30 values were >98.4% and GC contents were >51.95% (Table S2). The clean reads of each sample were mapped to the *S. scitamineum* genome at a range of 0.033% to 0.5738% (Table S2). These results suggested that the RNA-sequencing data were reliable and could be used for further analysis.

**Figure 2:**
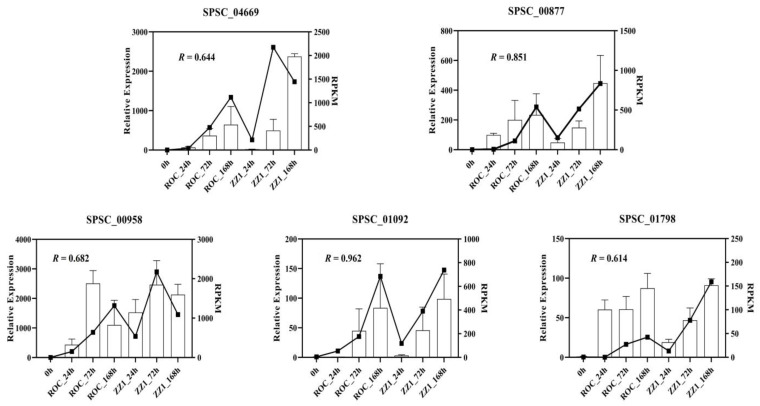
Validation of gene expression by RT-qPCR. Five randomly selected genes, SPSC_04669, SPSC_00877, SPSC_00958, SPSC_01092, and SPSC_01798, were selected for RT-qPCR analysis. Each treatment had three biological and three technical replicates. RNA-seq (left-axis) and RT-qPCR (right-axis) data representing the five genes are significantly correlated (*p* < 0.05).

After 24-h, 72-h, and 168-h colonization of sugarcane, *S. scitamineum*-infected ROC22 cultivars expressed 797, 2919, and 990 DEGs, respectively, while *S. scitamineum*-infected ZZ1 cultivars expressed 1226, 2421, and 1486 DEGs, respectively. Most DEGs (>70%) were upregulated when *S. scitamineum* was present. The amount of DEGs did not differ significantly between the ROC22 and ZZ1 cultivars (Table 1). Five genes were selected to validate the RNA-seq data. The RNA-seq and RT-qPCR data were significantly correlated with *R* values of 0.644, 0.851, 0.682, 0.962, and 0.614 (*p* < 0.05) for each of the five genes (Figure 2), indicating that the RNA-seq data were reliable.

### 3.3. Identification of Modules Associated with Infection

**Figure 3:**
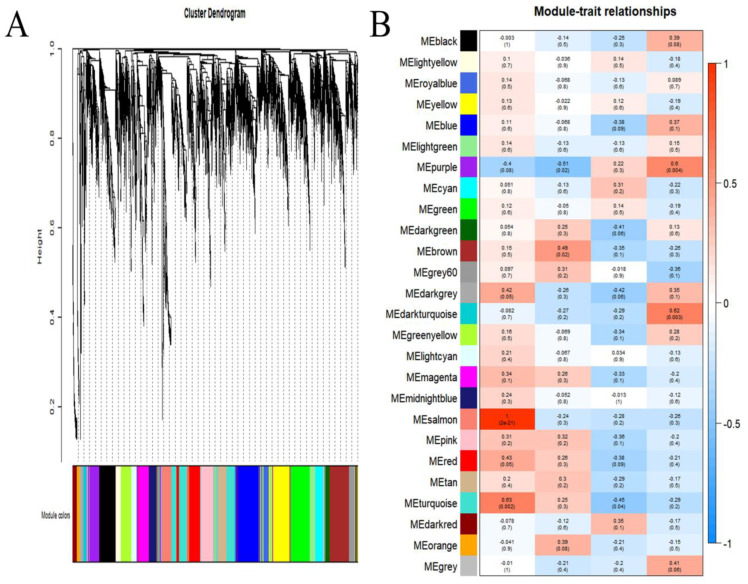
Co-expression network of traits related to *S. scitamineum* infection. (**A**) Co-expression gene modules of all expression profiles. A total of 26 distinct gene modules were identified. (**B**) Correlation of co-expressed gene modules and traits. The correlation coefficient and *p*-value are shown in each cell.

WGCNA was used to obtain gene modules exhibiting strong correlations with *S. scitamineum* in the plants. The expression profiles of all genes were used to construct a co-expression module in WGCNA. A total of 26 distinct gene modules were identified, and each module showed a specific expression pattern (Figure 3A). To identify gene modules related to *S. scitamineum* infection, a correlation analysis between traits and gene modules was conducted. The results indicated that the MEdarkturquoise and MEpurple modules were significantly and positively related to the *S. scitamineum* infection trait of 168 h (*r* = 0.62 and *p* = 0.003 for MEdarkturquoise and *r* = 0.6, *p* = 0.004 for MEpurple), and other modules were not significantly (*p* > 0.01) related to *S. scitamineum* infection traits (24 h, 72 h, and 168 h) (Figure 3B). Therefore, the genes in the MEdarkturquoise and MEpurple modules were selected for subsequent studies. There were 66 and 208 genes expressed in the MEdarkturquoise and MEpurple modules, respectively.

### 3.4. Annotation of Genes in the Modules

**Figure 4:**
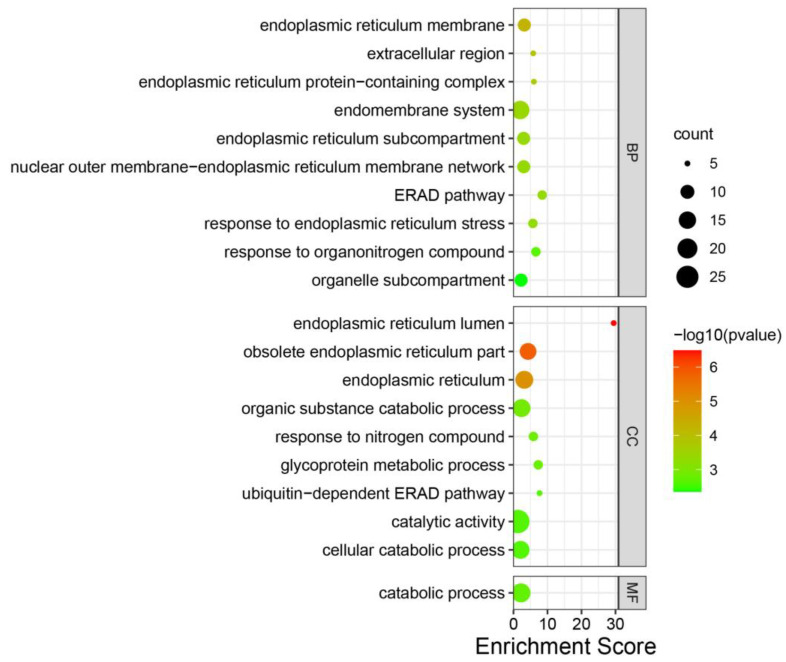
Enriched terms in MEpurple module identified by GO analysis. The columns and rows represent terms and enriched factors. The round size indicates the number of genes. BP, CC, and MF represent a biological process, cellular component, and biological process, respectively. A total of 20 GO terms were significantly enriched (*p* < 0.005).

To explore the function of the modules of interest, genes in each module were annotated using GO enrichment analysis. In the MEdarkturquoise module, no GO term enrichment was found. In the MEpurple module, twenty GO terms were significantly enriched (*p* < 0.005), including endoplasmic reticulum lumen, obsolete endoplasmic reticulum part, endoplasmic reticulum, endoplasmic reticulum membrane, extracellular region, endoplasmic reticulum protein-containing complex, endomembrane system, endoplasmic reticulum subcompartment, nuclear outer membrane–endoplasmic reticulum membrane network, ERAD pathway, response to endoplasmic reticulum stress, organic substance catabolic process, response to nitrogen compound, glycoprotein metabolic process, catabolic process, response to organonitrogen compound, ubiquitin-dependent ERAD pathway, catalytic activity, cellular catabolic process, and organelle subcompartment, which were related to the endoplasmic reticulum and catabolism (Figure 4, Table S3).

### 3.5. Hub Genes in the Modules

**Figure 5:**
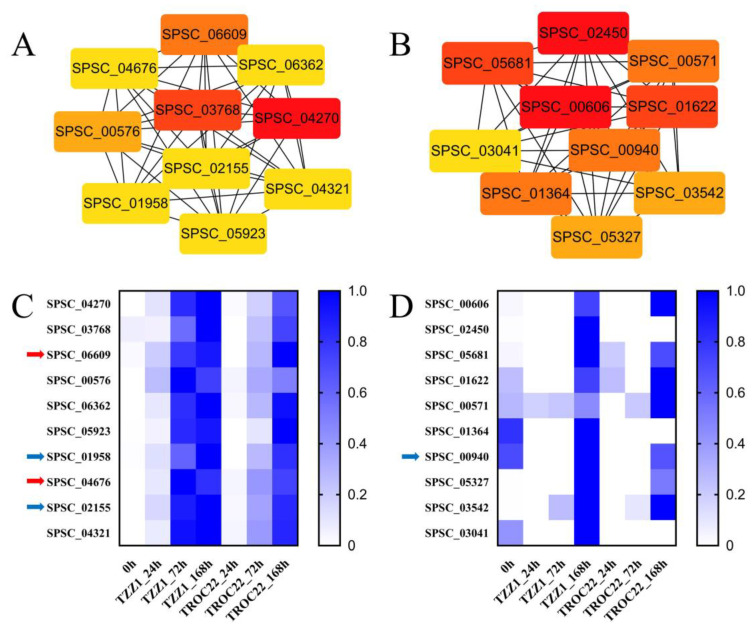
The top ten hub genes in the MEpurple and MEdarkturquoise modules. Co-expression networks of the top ten hub genes in the (**A**) MEpurple and (**B**) MEdarkturquoise modules and the details of hub genes were shown in Table 2. The hub genes were mined using the Cytohubba plugin in the Cytoscape v3.9.1 (Paul Shannon, Seattle, WA, USA). The cells are color-coded by degree (higher degree = cells with darker red color). Degree represents the number of connections at this gene according to co-expression analysis. Expression profiles of the top ten hub genes in the (**C**) MEpurple and (**D**) MEdarkturquoise modules. The RPKM were standardized by rows into fractions (0 to 1) for better visualization. The red arrows indicate the predicted small secreted cysteine-rich proteins (SCRPs). The blue arrows indicate proteins identified in the pathogen–host interactions (PHI) database.

The MEdarkturquoise and MEpurple modules were mined for hub genes that play an important role in *S. scitamineum* infection. Co-expression networks of the genes in these modules were separately constructed. According to the degree value, the top ten hub genes in each module were identified (Figure 5A,B). In the MEpurple module, five of the ten hub genes were annotated, and the gene with the highest value was SPSC_4270, which encoded the Mig1-Mig1 protein (Table 2). These genes exhibited higher expression with advanced infection stages (Figure 5C). In the MEdarkturquoise module, two of the ten hub genes were annotated, including transcription and mRNA export factor (SPSC_00571) and pleckstrin homology domain (Table 2). The expression levels of these genes increased with a longer *S. scitamineum* infection time. (Figure 5D).

### 3.6. Proteins in MEpurple and MEdarkturquoise Modules Related to Virulence

**Figure 6:**
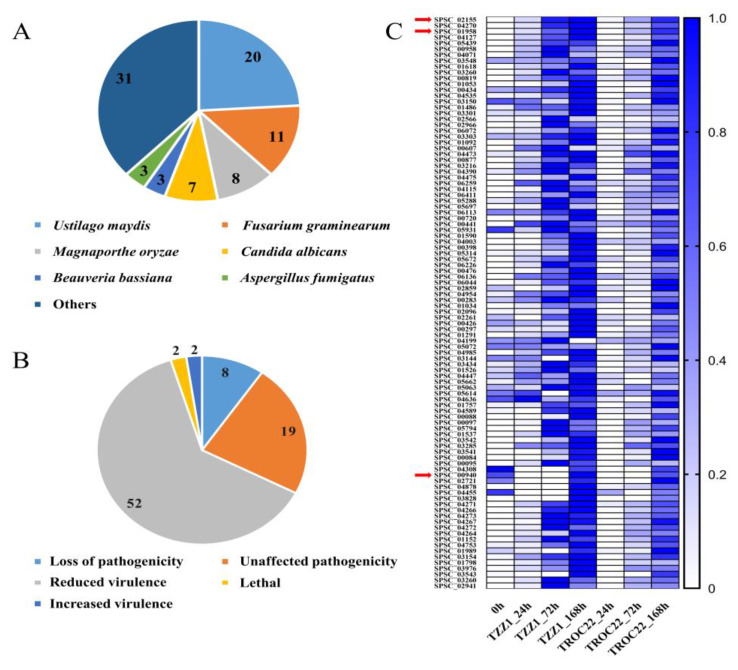
Virulence proteins identified in the PHI database. (**A**) Species distribution of homologs in the PHI database. (**B**) Virulence distribution of homologs in the PHI database. (**C**) Expression profiles of homologs during *S. scitamineum* infection. The RPKM were standardized by rows into fractions (0 to 1) for better visualization. The red arrows indicate hub genes identified by the Cytohubba plugin.

A total of 275 proteins in the MEpurple and MEdarkturquoise modules were blast against the PHI database to identify candidate proteins that may be associated with virulence. A total of 83 proteins with significant homology were identified (Table S4), of which 20 and 11 were from *Ustilago maydis* and *Fusarium graminearum* (Figure 6A), respectively. According to the PHI database, using gene deletion or silencing, 52 proteins exhibited reduced virulence, 19 proteins had no impact on pathogenicity, 8 proteins showed a loss of pathogenicity, and 2 proteins showed increased virulence (Figure 6B). The homologs of SPSC_02155, SPSC_03301, SPSC_04115, SPSC_06411, SPSC_00426, SPSC_01291, SPSC_01526, and SPSC_03828 showed a loss of pathogenicity and were annotated as a chorismate mutase, a secretory protein with a putative Ca^2+^ binding EF-hand motif, a heat shock protein in the Hsp40 group, a beta-1,6-glucan synthase, a DNA gyrase (bacterial topoisomerase II), a signal peptide peptidase, a CDK-related kinase, and a GTP-binding protein (Table S4), respectively. Interestingly, three proteins had homologs in the PHI database (Figure 5C,D), which may play an important role in *S. scitamineum* infection. The genes encoding these proteins were upregulated during disease and exhibited increased expression over time (Figure 6C). The functionally characterized effectors, *SsPep1* (SPSC_03260) and *SsPele1* (SPSC_02941), were also identified.

### 3.7. Candidate Effectors

**Figure 7:**
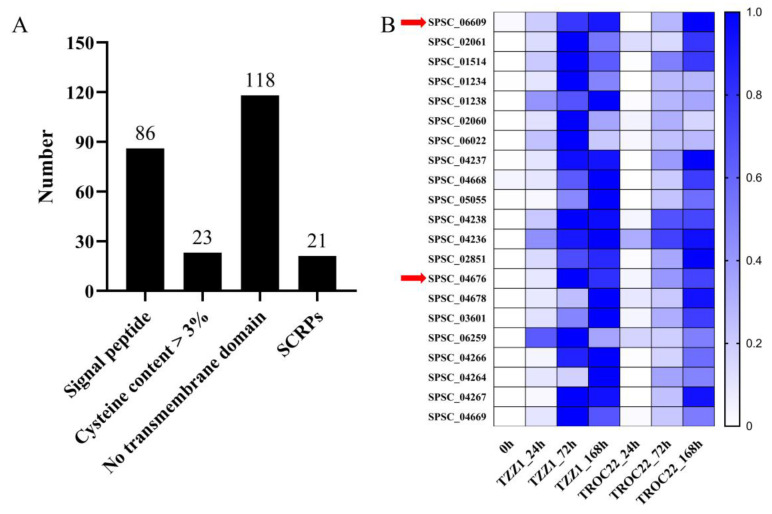
Small cysteine-rich proteins (SCRPs) in the MEpurple and MEdarkturquoise modules. (**A**) In 275 proteins from two modules, 86 had signal peptides, 23 had >3% cysteine content, and 118 had no transmembrane domain. A total of 21 proteins exhibited all these characteristics and were considered SCRPs. (**B**) Expression profiles of the predicted SCRP candidate effectors. The RPKM were standardized by rows into fractions (0 to 1) for better visualization. The red arrows indicate hub genes identified by the Cytohubba plugin.

The candidate effectors of *S. scitamineum* were predicted from 275 proteins in the MEpurple and MEdarkturquoise modules based on whether they had the characteristics of small secreted cysteine-rich proteins (SCRPs). A total of 121 proteins with a molecular mass of <400 aa were analyzed further. Of these, 23 proteins exhibited >3% cysteine content, 86 had a signal peptide (probability > 0.90), and 118 had no transmembrane domain (Figure 7A and Table S5). Based on SCRP characteristics that were previously described, 21 proteins were considered candidate effectors, of which 16 were annotated (Uniprot database) as uncharacterized while the rest were annotated as endoglucanases (SPSC_01514 and SPSC_06022) and Mig1 proteins (SPSC_04266, SPSC_04264 and SPSC_04267) (Table 3). Remarkably, two SCRPs, SPSC_04676 and SPSC_06609, were encoded by hub genes (Figure 5C), which may play a critical role in *S. scitamineum* virulence. The genes encoding these candidate effectors were upregulated during *S. scitamineum* infection and exhibited higher expression with longer *S. scitamineum* infection times (Figure 7B).

## 4. Discussion

### 4.1. WGCNA Is a Powerful Tool for Screening Modules and Hub Genes Related to Fungal Infection

WGCNA is an efficient method to reveal the correlation between gene modules and phenotypic traits [11,12]. In this study, only two gene modules (MEpurple and MEdarkturquoise) significantly associated with *S. scitamineum* infection (*p* < 0.01) were identified from WGCNA (Figure 3, Table 2), while other modules were not. Therefore, genes in the MEpurple and MEdarkturquoise modules were used for further analysis. Using the Cytohubba plugin, ten hub genes in each module were mined. In the MEpurple module, SPSC_04270, with the highest degree value, was annotated as the Mig1-Mig1 protein, which is involved in the formation of fungal hyphae and the regulation of genes encoding extracellular enzymes in *Saccharomycopsis fibuligera* [25]. Similarly, the Mig1 gene was upregulated during *S. scitamineum* infection [25]. SPSC_05923 and SPSC_01958 were annotated as glycosyl hydrolase and beta-N-acetylglucosaminidase, which are plant cell wall degradation enzymes that promote fungi infection and colonization [26,27]. SPSC_02155 encodes a chorismite mutase that is an essential effector in *Ustilago maydis* [28]. This gene was shown to be upregulated during *S. scitamineum* infection in a previous study [2]. In the MEdarkturquoise module, SPSC_00571 was annotated as a transcription and mRNA export factor, a protein required for exporting mRNAs from the nucleus to the cytoplasm [29], and SPSC_00940 was annotated as a pleckstrin protein that is involved in signal transduction [30]. Thirteen genes in the modules remain unannotated and will require further study to determine their functions.

### 4.2. Endoplasmic Reticulum and Catabolism Play Essential Roles in S. scitamineum Infection

In the current study, the MEpurple and MEdarkturquoise modules were associated with *S. scitamineum* infection using the WGCNA analysis. Of particular interest were genes in the MEpurple module that were enriched in endoplasmic reticulum-related terms (Figure 4). Endoplasmic reticulum is the main organelle involved in the secretion, folding, and assembly of membrane proteins [31]. When infecting plants, the endoplasmic reticulum of pathogenic fungi is involved in secreting cell wall hydrolases and multiple effectors in the plant cells that facilitate fungal colonization [4]. Furthermore, when pathogen fungi were *in planta*, they would be in the hypoxic host microenvironment and attacked by the host immune system, resulting in oxidative damage [32], which would trigger the unfolded protein response (UPR), thereby leading to the upregulated expression of endoplasmic reticulum-related genes in order to reduce damages and assist in the proper folding of proteins [33,34]. Thus, these two points implied that endoplasmic reticulum-related genes play a critical role in *S. scitamineum* infection in plants.

The fungi would secrete many enzymes to degrade the plant cell wall for colonizing host plants [35], such as endo-beta-1,4-xylanase [36] and laccase [37]. Genes in the MEpurple module were also enriched in catabolism-related GO terms (Figure 4); moreover, the genes, in catabolism-related GO terms, belong to plant cell wall degrading enzymes, implying that plant cell wall degrading enzymes would play an essential role in *S. scitamineum* infection. In the MEdarkturquoise module, no GO term enrichment was found, and some genes in the MEpurple module were not in enriched GO terms; in contrast maybe it’s because proteins encoded by these genes belonged to effectors, which are highly specific in different pathogenic fungi and can’t be annotated and enriched in GO analysis. Therefore, it was necessary to analyze these genes using other methods.

### 4.3. Mechanism for the Systematic Infection of Sugarcane Smut

The PHI database contains proteins that are functionally involved in pathogen–host interactions [38]. Through a homology search, 83 of 275 genes in the MEdarkturquoise and MEpurple modules had matches in the PHI database, 62 of which were assigned roles in fungal virulence (Table S4). Notably, three proteins, SPSC_01958, SPSC_02155, and SPSC_00940, were encoded by hub genes (Figure 5C,D). Furthermore, two functionally characterized effectors of *S. scitamineum*, *SsPep1* (SPSC_03260) and *SsPele1* (SPSC_02941) [7,39], belonged to MEpurple modules, suggesting that there is a reliable association between MEpurple modules and *S. scitamineum* infection.

Candidate *S. scitamineum* effectors were predicted in the genes under the MEpurple and MEdarkturquoise modules. SCRPs are a kind of effector that can either activate host resistance or increase host susceptibility [7,39]. The disulfide bond formed by cysteine helps to keep effectors stable in the host environment [40]. In this study, 21 proteins had SCRPs characteristics (Figure 7), of which the novel candidate effectors, SPSC_04676 and SPSC_06609, were encoded by hub genes, implying they played an important role in *S. scitamineum* infection.

**Figure 8:**
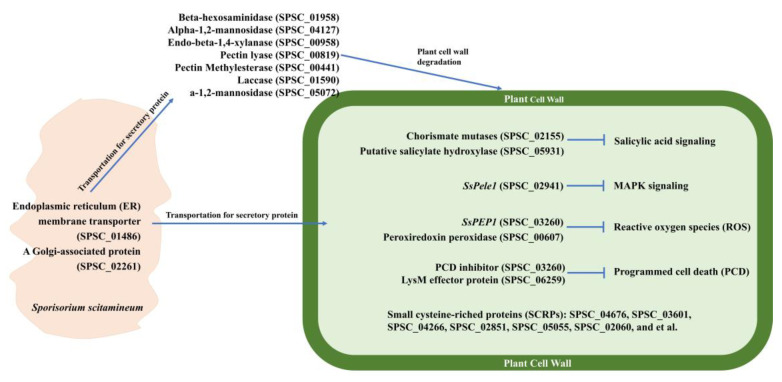
A working model of *S. scitamineum* proteins in infection. The model was postulated based on current findings combined with the results of PHI database BLASTp and SCRPs prediction in MEpurple and MEdarkturquoise modules. The representative proteins with different mechanisms of *S. scitamineum* infection were chosen in this model. The expression profiles of these genes were shown in Figure 6 and Figure 7. The functions of the listed proteins were assumed by annotation. The functions of the predicted SCRPs remained unknown.

Sugarcane smut is a systemic disease in which the pathogenic fungus is completely embedded in the host plant, a hostile environment for the pathogen [40]. To assure its colonization, the smut fungus has developed strategies to avoid host attack by secreting effectors that will suppress the host immune response [5]. The limited number of hydrolases and large number of effectors/candidate effectors identified in the modules correlates with the systemic nature of *S. scitamineum* infection and the need to both avoid pathogen-triggered immunity (PTI) and suppress effector-triggered immunity (ETI). Current findings combined with the results of PHI database BLASTp and SCRPs prediction in the MEpurple and MEdarkturquoise modules led us to postulate a *S. scitamineum* infection model. Firstly, endoplasmic reticulum and Golgi-related proteins would assist in the transport of proteins that are involved in virulence. Secondly, the secreted enzymes would degrade plant cell wall for *S. scitamineum* colonization. The secreted effectors would interfere with different physiological functions of sugarcanes, including signal transduction, reactive oxygen species (ROS), and programmed cell death (PCD) (Figure 8).

In *Ustilago maydis*, the chorismate mutase could converse the shikimic acid to aromatic amino acids instead of salicylic acid, which would reduce the salicylic acid content *in planta*, affecting the salicylic acid signal pathway and causing a decrement of host defense response [41]. The *S. scitamineum* homolog, SPSC_02155, of *Ustilago maydis* chorismate mutase may affect the salicylic acid pathway in sugarcane and decrease the host defense response through the same mechanism. As for *Sspele1* (SPSC_02941), it is a *S. scitamineum* effector that could bind to the extracellular domain of *ScPEPR1* to suppress the *ScPEPR1*-mediated MAPK signaling pathway [7]. The oxidative stress is one of the mechanisms of sugarcane response to *S. scitamineum* infection [18], and the *S. scitamineum* effector *SsPEP1,* could inhibit peroxidase, thereby suppressing the sugarcane defense against *S. scitamineum* infection [39]. Programmed cell death (PCD) of plants is a strategy that could prevent pathogenic fungi infection [42]. The *U. maydis* effector of the PCD inhibitor could inhibit PCD during infection [43], indicating that the *S. scitamineum* homolog, SPSC_03260, may also inhibit sugarcane PCD. In *Verticillium dahlia*, the SCRPs could activate or inhibit host ETI [21,44,45], which play important roles in *V. dahlia* infection. The functions of predicted *S. scitamineum* SCRPs may play similar roles as *V. dahlia* SCRPs, and function characterization of these SCRPs will be conducted in a future study.

## 5. Conclusions

Two gene modules, containing a total of 275 genes, significantly associated with *S. scitamineum* infection were identified using WGCNA (*p* < 0.01). GO enrichment analysis of these genes revealed that endoplasmic reticulum-related and catabolism-related genes were implicated in *S. scitamineum* infection. The top 10 hub genes in each module were identified using the Cytohubba plugin. Of 275 genes, 96 genes were identified in the PHI database, 3 of which belonged to hub genes, and 21 genes encoding proteins showed SCRPs characteristics that were considered as candidate effectors—2 of which belonged to hub genes. A *S. scitamineum* infection model was postulated based on current findings. These findings help to deepen the current understanding of early events in *S. scitamineum* infection.

## Figures and Tables

**Table 1:** Statistics of differentially expressed genes.

Group	Upregulated	Downregulated	All
TROC22_24 h vs. 0 h	763	34	797
TROC22_72 h vs. 0 h	2887	32	2919
TROC22_168 h vs. 0 h	705	285	990
TZZ1_24 h vs. 0 h	896	330	1226
TZZ1_72 h vs. 0 h	2240	181	2421
TZZ1_168 h vs. 0 h	1225	261	1486

**Table 2:** Top ten hub genes in the MEpurple and MEdarketurquoise modules.

Gene ID	Gene Module	Description	Degree	CDS Length
SPSC_04270	MEpurple	Mig1-Mig1 protein	42	203
SPSC_03768	MEpurple	Uncharacterized protein	36	571
SPSC_06609	MEpurple	Uncharacterized protein	35	133
SPSC_00576	MEpurple	Uncharacterized protein	34	403
SPSC_06362	MEpurple	Uncharacterized protein	33	1745
SPSC_05923	MEpurple	Glycosyl hydrolase	33	737
SPSC_01958	MEpurple	Beta-N-acetylglucosaminidase	33	703
SPSC_04676	MEpurple	Uncharacterized protein	33	180
SPSC_02155	MEpurple	Secreted chorismate mutase	33	303
SPSC_04321	MEpurple	Collagen	33	584
SPSC_00606	MEdarkturquoise	Uncharacterized protein	17	125
SPSC_02450	MEdarkturquoise	Uncharacterized protein	17	75
SPSC_05681	MEdarkturquoise	Uncharacterized protein	16	911
SPSC_01622	MEdarkturquoise	Uncharacterized protein	16	300
SPSC_00571	MEdarkturquoise	Transcription and mRNA export factor	14	113
SPSC_01364	MEdarkturquoise	Uncharacterized protein	14	118
SPSC_00940	MEdarkturquoise	Pleckstrin homology domain protein	14	1428
SPSC_05327	MEdarkturquoise	Uncharacterized protein	13	167
SPSC_03542	MEdarkturquoise	Uncharacterized protein	13	370
SPSC_03041	MEdarkturquoise	Uncharacterized protein	12	342

Note: Degree represents number of connections at this gene according to co-expression analysis.

**Table 3:** Prediction of candidate effectors in *S. scitamineum*.

Gene ID	Uniprot Annotation	Length (AA)	Cysteine Percentage (%)	Signal Peptide Likelihood	Signal Peptide Position	Location	Transmembrane Domain Numbers
SPSC_06609	Uncharacterized protein	133	6.0150	0.9998	pos: 26–27	Extracellular (Secreted)	0
SPSC_02061	Uncharacterized protein	157	5.0955	0.9997	pos: 23–24	Extracellular (Secreted)	0
SPSC_01514	Related to Endoglucanase 1	311	4.8232	0.9997	pos: 29–30	Extracellular (Secreted)	0
SPSC_01234	Uncharacterized protein	136	4.4118	0.9998	pos: 24–25	Extracellular (Secreted)	0
SPSC_01238	Uncharacterized protein	144	4.1667	0.9998	pos: 21–22	Extracellular (Secreted)	0
SPSC_02060	Uncharacterized protein	194	4.1237	0.9998	pos: 25–26	Extracellular (Secreted)	0
SPSC_06022	Probable Endoglucanase 1	380	3.9474	0.9998	pos: 26–27	Extracellular (Secreted)	0
SPSC_04237	Uncharacterized protein	130	3.8462	0.9997	pos: 21–22	Extracellular (Secreted)	0
SPSC_04668	Uncharacterized protein	132	3.7879	0.9997	pos: 24–25	Extracellular (Secreted)	0
SPSC_05055	Uncharacterized protein	80	3.7500	0.9970	pos: 32–33	Extracellular (Secreted)	0
SPSC_04238	Uncharacterized protein	136	3.6765	0.9998	pos: 22–23	Extracellular (Secreted)	0
SPSC_04236	Uncharacterized protein	229	3.4934	0.9998	pos: 21–22	Extracellular (Secreted)	0
SPSC_02851	Uncharacterized protein	292	3.4247	0.9998	pos: 26–27	Plasmamembrane	0
SPSC_04676	Uncharacterized protein	180	3.3333	0.9997	pos: 23–24	Extracellular (Secreted)	0
SPSC_04678	Uncharacterized protein	150	3.3333	0.9998	pos: 24–25	Extracellular (Secreted)	0
SPSC_03601	Uncharacterized protein	181	3.3149	0.9997	pos: 20–21	Extracellular (Secreted)	0
SPSC_06259	Uncharacterized protein	302	3.3113	0.9998	pos: 20–21	Nuclear	0
SPSC_04266	Related to Mig1 protein	185	3.2432	0.9998	pos: 25–26	Extracellular (Secreted)	0
SPSC_04264	Related to Mig1 protein	191	3.1414	0.9998	pos: 27–28	Extracellular (Secreted)	0
SPSC_04267	Related to Mig1 protein	197	3.0457	0.9998	pos: 27–28	Extracellular (Secreted)	0
SPSC_04669	Uncharacterized protein	132	3.0303	0.9997	pos: 24–25	Extracellular (Secreted)	0

Note: These proteins were considered candidate effectors based on the characteristics of small secreted cysteine-rich proteins (SCRPs). Other prediction results are listed in Table S4.

## Data Availability

The raw sequence data reported in this paper have been deposited in the Genome Sequence Archive in National Genomics Data Center, China National Center for Bioinformation/Beijing Institute of Genomics, Chinese Academy of Sciences (GSA: CRA006643) and are publicly accessible at https://ngdc.cncb.ac.cn/gsub/ (accessed on 5 July 2022).

## Supplementary Materials

The following supporting information can be downloaded at: https://www.mdpi.com/article/10.3390/jof8080852/s1, Figure S1: Standard curve of TaqMan RT-qPCR; Table S1: RT-qPCR primers used in the study; Table S2: Statistics of RNA sequencing data; Table S3: GO enrichment analysis in MEpurple module; Table S4: BLASTp results of genes in the MEpurple and Medarkturquoise modules against the PHI database; Table S5: Prediction of candidate effectors in *S. scitamineum*.
