# Supplementary material for: Identification of Gene Modules and Hub Genes Associated with Sporisorium scitamineum Infection Using Weighted Gene Co-Expression Network Analysis
Source: J Fungi (Basel). 2022 Aug 15;8(8):852. doi: 10.3390/jof8080852 (PMC9409688; doi:10.3390/jof8080852)
Supplement: Supplementary file 1 [file jof-08-00852-s001.zip › Supplementary File 1.pdf]

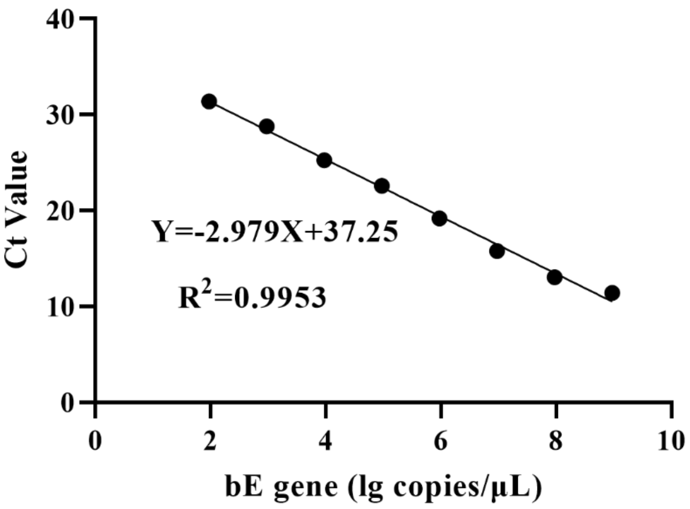

Figure S1. Standard curve of TaqMan RT-qPCR. The Ct values ranged from 11.41 to 31.39. The lowest concentration of the bE gene standard was 94.65 copies/μL. The amplification efficiency was 1.16 according to the equation: E = 10(-1/Slope)-1, and R^2 = 0.9953. The standard curve was reliable and was used for further analysis.
